# Smart Monitoring for Cancer Treatment: Feasibility Study of an IoT-Based Assessment System

**DOI:** 10.3390/s26051579

**Published:** 2026-03-03

**Authors:** David Martínez-Pascual, Pablo Rubira-Úbeda, José M. Catalán, Andrea Blanco-Ivorra, Beatriz Franqueza, Gabrielle Derrico, Juan A. Barios, Nicolás García-Aracil

**Affiliations:** 1 Robotics and Artificial Intelligence Group, Bioengineering Institute, Miguel Hernández University, 03202 Elche, Alicante, Spain; 2 Innovative Devices for Rehabilitation and Assistance (iDRhA), 03202 Elche, Alicante, Spain; pablo.rubira@goumh.umh.es; 3 Bienzobas-Atrys, 04001 Almería, Almería, Spain; 4 Hospital de San Juan de Alicante, 03550 Alicante, Alicante, Spain; barios_jua@gva.es

**Keywords:** wearable sensors, Internet of Things, remote patient monitoring, machine learning

## Abstract

Non-invasive monitoring technologies are increasingly being explored to support cancer care, yet most existing approaches focus on isolated parameters and fail to provide a comprehensive view of patients’ health. This study presents a feasibility evaluation of an IoT-based system designed to detect treatment-related problems in oncology patients through the integration of wearable sensors, physiological measurements, and patient-reported outcomes. A monitoring kit, including a smartwatch, tensiometer, weighing scale, and mobile device, was deployed in a cohort of 26 patients undergoing oncological treatment. Data acquisition followed a structured schedule: continuous physiological recording via the smartwatch, daily blood pressure measurements, weekly weight monitoring, and structured surveys capturing treatment-related side effects. These heterogeneous data were transformed into binary clinical metrics using rule-based feature extraction algorithms, covering conditions such as insomnia, nausea, diarrhea, abdominal pain, headache, weight loss, hypertension, and fever. Clinical specialists labeled the dataset to ensure medical validity. Machine Learning models were then trained to analyze the features and generate alerts for potential treatment complications. The results demonstrate the feasibility of integrating IoT and Artificial Intelligence techniques for continuous, patient-centered monitoring in oncology, paving the way for intelligent decision-support systems that enhance early detection and clinical management.

## 1. Introduction

Recent advancements in monitoring technology, particularly through the use of wearable devices and Internet of Things (IoT) devices, are generating significant expectations for improving the care of cancer patients and enhancing cancer research. These devices can measure various parameters that impact the health of these patients [1]. In healthcare, the IoT consists of an interconnected ecosystem of medical devices, sensors, software applications, and systems that collect, transmit, and analyze health data [2]. It is noteworthy that the foundation of any IoT health system is its sensor layer. This layer includes a variety of devices designed to capture specific health metrics. In oncology, this can consist of standard sensors for monitoring vital signs, such as Heart Rate (HR), Blood Pressure (BP), and body temperature [3]. By leveraging these technologies, remote patient monitoring (RPM) platforms can transcend the traditional limitations of distance and time, bringing healthcare services directly to patients’ homes [4,5]. This approach enables continuous data collection, providing a comprehensive and detailed report of patients’ health status that would not be possible to obtain through intermittent in-clinic visits.

Current scientific evidence supports the viability and benefits of using mobile technology and wearable sensors for remote monitoring to improve outcomes in cancer patients during and after treatment. Diverse studies have shown the feasibility of using IoT monitoring together with mobile platforms for patients undergoing chemotherapy or radiotherapy to collect data on their quality of life [6,7]. These studies indicated a high level of acceptability and engagement with the technology, as shown by high completion rates of patient-submitted monitoring reports through mobile applications and the use of monitoring devices. These positive feasibility results are an essential first step toward promoting research and implementing these tools in future clinical care. Furthermore, randomized trials showed that clinical and remote monitoring of symptoms during chemotherapy resulted in improved quality of life, fewer treatment interruptions, and improved survival rates in cancer patients, regardless of whether they had metastatic disease [8,9].

The data collected through these systems can be used to generate timely alerts for healthcare providers, facilitating early interventions and better management of treatment-related side effects [10]. It is worth mentioning that generating alerts for clinicians requires analyzing the collected data. A primary function of Machine Learning (ML) models in RPM is to transform raw data into valuable information for clinicians. These systems act as tools that analyze immense volumes of data from multiple sources to identify patterns and predict potential health issues before they become critical [11]. By processing this information, the system can generate clinically actionable insights and alerts, which allows the care team to proactively adjust treatment plans and implement early interventions [12].

However, a major challenge for developing robust ML models in healthcare is the limited availability of high-quality, large datasets [13]. In this context, synthetic data generation offers a powerful solution to this problem by creating artificial datasets that replicate the statistical properties and inter-variable relationships of real patient data without containing any identifiable information. The primary advantage of this method is the mitigation of privacy risks, which traditionally restricts access to sensitive medical records and hinders collaborative efforts. With synthetic data, it becomes possible to publicly release datasets for tasks like model benchmarking and external validation, accelerating the pace of innovation. This is particularly crucial for improving model performance on underrepresented patient populations or rare conditions, where real-world data are inherently scarce, allowing for the creation of more equitable and effective healthcare algorithms [14].

This work presents a feasibility study on an intelligent monitoring system designed for oncological patients. The system is composed of different IoT and wearable devices that allow for capturing physiological measures during patients’ daily life settings. In addition, self-reported effects of oncological treatments are collected by means of surveys. Diverse metrics are extracted from the recorded physiological signals and surveys, which are then used to train ML models to generate alerts regarding the patient’s oncological treatment tolerance. Furthermore, a rule-based parametric simulation model is proposed to generate synthetic data to develop a robust ML model. These data are used together with data collected from 26 people undergoing oncological treatment who used the presented IoT-based monitoring system.

## 2. Related Works

RPM has been utilized since the early 2000s [15]. RPM emerged as an effective approach to address the challenges faced by patients experiencing prolonged periods without clinical supervision, and its significance was further underscored during the COVID-19 pandemic, which necessitated remote medical oversight to mitigate infectious risk. RPM applications span a variety of medical domains, including heart failure [16], chronic kidney disease [17], geriatric care [18], and oncology [19]. Cumulative evidence demonstrates the benefits of RPM. In [8,16], the authors affirm that it improves quality of life and reduces hospital admissions. In other studies [18], it is concluded that hospital stays are shorter when combined with RPM, and even in oncology, the results have shown that patient survival may be extended by up to five months.

An initial attempt at a comprehensive implementation is described in [2], in which a network of body sensors was employed to collect diverse physiological data such as electrocardiogram (ECG), BP, HR, and body temperature. These sensors automatically upload data to the cloud, eliminating the need for an intermediate node. However, this study does not incorporate any algorithms or ML techniques to enable automatic assessment of the patient’s clinical status.

Several studies have proposed end-to-end pipelines, as exemplified by [20], which presents an integrated BP telemonitoring system. The primary objective of this study is to utilize ML algorithms for real-time classification of BP measurements. The system not only measures BP but also classifies results and transmits, stores, and displays the data, providing a comprehensive telemonitoring solution. A comparable approach is reported in [21], where ECG data are acquired via a wearable node. Signal conditioning is performed using high-frequency noise reduction and baseline correction through polynomial interpolation, followed by feature extraction of the R wave from the QRS complex using a Multilayer Perceptron Neural Network. Nevertheless, these studies do not account for the patient’s overall health status, instead concentrating on isolated metrics or specific physiological parameters. This methodology, while effective for targeted clinical outcomes, neglects the intricate relationships among physiological signals, treatment-related adverse events, and patients’ subjective experiences. Consequently, such systems are constrained in their ability to provide a comprehensive assessment of the patient’s condition during therapy.

Other works aim to determine more complex metrics like stress that manifest through multiple physiological variables. In [22], a system based on ML is presented to monitor a patient’s stress level and classify it into three levels: low, normal, and high. To achieve this, the monitored biological data include body temperature, humidity, and patient activity (steps), all collected through a wearable bracelet. The proposed model does not integrate other relevant factors that may influence stress, such as the patient’s medical history, age, weight, gender, or environmental factors.

Another problem is pointed out in [17,19], which is the low adherence of patients to wearable devices. This is a critical factor, as the clinical utility of any RPM system hinges entirely on the patient’s willingness to consistently use the monitoring technology. Poor adherence directly leads to gaps in data collection, potentially masking periods of clinical deterioration and undermining the system’s predictive capabilities.

In [23], the primary objective is to develop an RPM system using IoT sensors to collect patient vital signs such as body temperature, ECG, BP, breathing rate, oxygen saturation, and activities in real time. This system uses an ML model to accurately recognize human activities and automatically alert medical personnel or family members in cases of health emergencies or quarantine breaches. However, this work focuses only on suspected COVID-19 patients, not oncology patients or patients undergoing treatment.

In the field of oncology, the study performed in [9] aimed to evaluate the impact of electronic patient-reported symptom monitoring on overall survival among patients undergoing routine chemotherapy for metastatic solid tumors. This randomized clinical trial employed a web-based questionnaire platform, enabling patients to self-report 12 common symptoms. Reports of severe or worsening symptoms triggered automated email alerts to a clinical nurse, facilitating prompt intervention. The hypothesized mechanism underlying this benefit is the enhanced responsiveness to patient-reported symptoms, which may prevent adverse sequelae and enable patients in the intervention group to tolerate chemotherapy for longer durations. However, this work focuses only on patients’ self-reports of symptoms, without extracting or integrating any objective biological metrics or physiological sensor data to provide a more comprehensive and objective view of the patient’s condition.

The unique contribution of this work lies in the architectural design and clinical integration of an IoT-based system specifically engineered to assess treatment tolerance in oncology patients by combining continuous physiological monitoring, structured self-reports, and ML-driven analytics. Unlike existing remote monitoring solutions that often focus on isolated parameters or high-level wellness metrics, our workflow utilizes a patient-friendly monitoring kit (comprising a smartwatch, tensiometer, and digital scale) to enable the daily capture of diverse data streams. This hardware setup is complemented by a mobile platform with structured instructions that guide patients to report blood pressure, weight, and treatment-related side effects through tailored surveys, ensuring high data quality and adherence. By mapping these integrated signals directly to clinically grounded thresholds for treatment tolerance, the system bridges the gap between raw sensor data and actionable medical insights. Furthermore, the introduction of a rule-based parametric simulation framework to generate synthetic data addresses the bottleneck of low event prevalence in pilot clinical trials. This dual-layer approach, which utilizes real-world patient data to anchor clinical patterns and synthetic data to expand the model’s predictive reach, demonstrates a scalable pathway for developing early-warning systems capable of anticipating treatment-related complications before they necessitate emergency intervention.

## 3. Materials and Methods

### 3.1. Participants

A total of 26 oncology patients were included from three hospital centers: Madrid, Almería, and Córdoba. The sex distribution was balanced, with 51.1% being male and 48.9% being female. Patient ages ranged from 44 to 85 years, with a mean age of 64 years.

Regarding functional status, most patients had an ECOG performance status of 0 (93.3%), while a minority had ECOG 1 (6.7%). The most frequent clinical stage was stage IV (62.2%), followed by stages III (24.4%), II (11.1%), and I (2.2%).

Inclusion criteria required that patients were undergoing oncological treatment or in a therapeutic break, and were capable of operating the monitoring devices. Although patients undergoing radiotherapy were initially considered, the sample size in this subgroup was insufficient, and, therefore, they were excluded from the final analysis.

Patient recruitment was carried out by the oncologists at each center during routine clinical follow-up. All patients were informed about the objectives of the study and signed the informed consent form prior to inclusion. The research team provided technical support for device management. The study was approved by the Ethics Committee for Research with Medicines (CEIm) of HM Hospitales (approval code: 24.01.2276-GHM). All participants provided written informed consent prior to their inclusion in the study. The research was conducted in accordance with ethical principles and current data protection regulations. Data collection and processing were carried out according to GDPR through pseudonymization.

A 3-week minimum monitoring period was proposed by the research team, and the research team provided technical support for device management. The mean recording period was 6.5 weeks. Neither missing data nor survey incompletion was reported during the monitoring periods for any of the users.

### 3.2. IoT-Based Monitoring System

The patient monitoring stage is responsible for the acquisition, collection, and cloud storage of raw data. Data were transmitted from the wearable kit to the mobile device via Bluetooth using standard encryption protocols, while all cloud-based transfers were secured using HTTPS/TLS and stored in a cloud infrastructure based on Microsoft Azure (Microsoft Corporation, Redmond, WA, USA). A smartwatch (Fitbit Sense 2, Fitbit Inc., San Francisco, CA, USA), a weight scale (OMRON Viva, OMRON Healthcare Co., Ltd., Kyoto, Japan), and a BP monitor (OMRON M4 Intelli IT, OMRON Healthcare Co., Ltd., Kyoto, Japan) were used as the devices in the RPM system. To provide the feature extraction stage with the necessary inputs for deriving binary features, multiple biological variables are measured across different health domains, including thermoregulatory signals, physical activity, sleep quality, metabolic indicators, and cardiovascular parameters. In addition, subjective information is gathered through patient-reported data to capture individual perceptions and treatment-related experiences. To obtain these measurements, patients are provided with a wearable device kit consisting of a smartwatch, a weighing scale, a tensiometer, and a mobile device. The selection of the metrics across the established health domains is supported by clinical evidence.

Continuous monitoring of Skin Temperature provides vital surveillance for systemic changes, enabling the derivation of approximate fever thresholds critical for early detection. This is particularly important for febrile neutropenia, an oncological emergency, formally defined by clinical guidelines [24]. While skin temperature is not a direct core temperature measurement, its continuous trend data and established correlation allow it to function as a surrogate for identifying a significant temperature elevation warranting clinical follow-up [25].

In addition, monitoring the HR and daily BP measurements provides essential physiological context for evaluating cardiovascular load, autonomic regulation, and overall systemic homeostasis.

Wearable-derived metrics for Daily Activity and Sleep-Log are strong, objective indicators of patient functional status and energy levels. Fatigue and functional decline are among the most common and prognostic side effects of cancer treatment. Tracking these variables provides objective evidence of morbidity, helping clinicians distinguish between routine tiredness and clinically significant decline in performance status [26].

Monitoring weight is a fundamental component of cancer care. Unintended weight change is a critical indicator of nutritional status, potential cancer cachexia, and malabsorption. Weekly weighing is essential for early identification of malnutrition, which can severely impact treatment tolerance and overall prognosis [27].

In addition, self-reporting adverse effects via surveys is indispensable, as these metrics capture the patient’s subjective experience of adverse effects (e.g., gastrointestinal issues, pain, alopecia). A clinical trial demonstrated that self-reported adverse effects during chemotherapy leads to improved overall survival and quality of life by enabling timely clinical intervention before symptoms escalate [9].

Data acquisition follows a structured schedule designed to balance patient convenience with the collection of a robust and comprehensive dataset. The smartwatch continuously records the physiological parameters listed in Table 1 throughout the day with no required patient intervention. BP measurements are taken once daily using the tensiometer, while the weighing scale is used weekly to track changes in body mass. The indication to perform the measurements comes from the mobile device, which serves as the central hub for patient interaction, user interface and local server.

**Figure 1:**
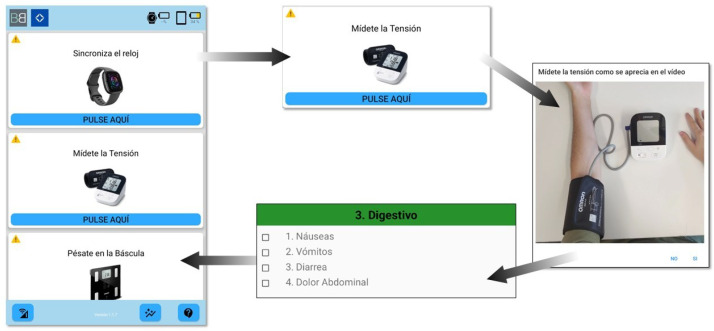
Pipeline of a measurement example process.

The mobile device hosts an application that acts as a virtual assistant, reminding patients when to perform their measurements and guiding them through the entire process, as shown in Figure 1. Once per day, the application issues an alert prompting the synchronization of smartwatch data (done automatically), as well as the acquisition of BP and weight measurements. Upon selecting a task, the application provides step-by-step instructions, including demonstration videos and detailed guidance, to ensure correct measurement execution. After completing the measurement routine, patients are asked to complete a survey designed to capture subjective health information. This survey is structured into four categories: physical activity, digestive symptoms, pain, and other, enabling a comprehensive assessment of the patient’s condition.

All recorded and self-reported data are securely uploaded to a cloud-based storage system once daily, ensuring timely availability for further processing. This continuous data collection forms the foundation for the subsequent feature extraction stage, where raw measurements are transformed into binary features that are fed into the ML pipeline for clinical decision support.

### 3.3. Feature Extraction from Data Collected

Following acquisition of raw data from wearable devices and patient-reported surveys, the subsequent process involves transforming these heterogeneous signals into clinically interpretable metrics. The process of transforming heterogeneous raw signals into discrete binary features represents a necessary methodological simplification designed to bridge the gap between high-frequency data and actionable clinical insights. By aggregating continuous physiological recordings and daily patient-reported outcomes into weekly binary metrics, the system focuses on identifying persistent clinical states rather than transient fluctuations.

Each condition is inferred using a combination of objective physiological indicators and subjective data obtained from patient surveys. Individual baseline values are established for each patient, and deviations exceeding clinically validated thresholds result in the generation of binary metrics (presence or absence) for each condition. These binary features yield standardized and comparable inputs suitable for training ML models dedicated to problem detection and clinical decision support.

#### 3.3.1. Insomnia

Insomnia is a common sleep disorder characterized by difficulty falling asleep, staying asleep, or waking up too early and not being able to get back to sleep. As a result, individuals may experience daytime fatigue, mood disturbances, and impaired cognitive function [28]. Insomnia is considered possible if at least two of the following conditions are met for a period of at least 3 consecutive nights (or 4 out of 7 nights in a week). The baseline period for every patient is seven days:

1. Reduced Sleep Duration. Total nocturnal sleep duration is lower than baseline by a predefined threshold (>20–30 % reduction) or less than 6 h [29].
2. Decreased Sleep Efficiency. Sleep efficiency is lower than baseline (by a threshold >10–15% reduction) or less than 80–85% in absolute value [29,30].
3. Sleep Fragmentation (Stages). Reduction of deep sleep and/or REM sleep compared to baseline (15–20% reduction).

#### 3.3.2. Abdominal Pain, Cephalea, Nausea, Vomits and Diarrhea

Abdominal pain, cephalea (headache), nausea, vomits and diarrhea are common adverse effects in oncology patients that can significantly affect quality of life. Unlike other conditions, such as insomnia or hypertension, which can be inferred from physiological parameters, these symptoms cannot be reliably detected through wearable devices. Instead, they are assessed directly through patient self-reporting.

For all conditions, the detection method relies exclusively on survey responses. If the patient indicates the presence of either symptom in the corresponding section of the survey, the binary metric is marked as positive; if no symptom is reported, the metric remains negative. This approach ensures that these subjective experiences, which are best captured through direct patient feedback, are systematically incorporated into the dataset while maintaining consistency with the binary feature extraction framework.

#### 3.3.3. Weight Loss

Unintentional weight loss is a critical sign in oncology patients. International guidelines define cancer cachexia as a loss of >5% of body weight over 6 months (or >2% if the initial BMI is <20) [31,32].

In this case, only a 6-week follow-up period is available. Therefore, it is necessary to proportionally adjust the thresholds based on the criterion of 5% over 6 months. In absolute terms, 5% over 26 weeks corresponds to approximately 1.15% over 6 weeks. Thus, a threshold of approximately 1–2% weight loss in 6 weeks can be considered an early warning. In patients with a BMI < 20, a 2% loss over 6 months corresponds to approximately 0.5% over 6 weeks (although in practice, due to measurement noise, this may be rounded to 1%). While there is no official guideline for such short periods, this linear scaling represents the most direct approximation.

Some studies [33,34] recommend consulting a physician if more than 5% of body weight is lost in one year, suggesting that even a 1–2% loss in 6 weeks could be clinically significant.

To monitor weight changes, a baseline is first established by averaging the initial weekly measurements at the beginning of the observation period. Following this, the current weight is recorded weekly using a smart scale. The percentage variation (Δ%) from the baseline is calculated for each new measurement:

(1) $$\Delta \%=\frac{\mathrm{Baseline}\;\mathrm{Weight}-\mathrm{Current}\;\mathrm{Weight}}{\mathrm{Baseline}\;\mathrm{Weight}}\times 100.$$

This calculated Δ% is then compared against adapted thresholds to detect significant weight loss. Over a 6-week period, a Δ% of approximately 1–2% or greater is considered equivalent to the clinical criterion of 5% weight loss over 6 months. For participants with an initial BMI below 20, a more sensitive threshold of approximately 0.5–1% is utilized.

To determine the presence of involuntary weight loss, it will be verified whether Δ% has exceeded the threshold during the measurement period, thus indicating possible cachexia in accordance with clinical criteria.

#### 3.3.4. Hypertension

Hypertension is defined as chronically elevated BP. In oncology patients, it may represent a preexisting comorbidity or an adverse effect of certain treatments. Its value is obtained directly from the BP monitor, and standardized thresholds are used to determine the condition in adults. According to European guidelines, hypertension is defined as BP ≥140/90 mmHg [35], while other guidelines use a threshold of 130/80 mmHg.

Hence, if both systolic and diastolic BP values exceed the recommended thresholds across multiple measurements, the condition of hypertension is determined.

#### 3.3.5. Pyrexia

Fever is defined as an elevation of core body temperature above the normal range (>37.8 °C) [25]. In oncology patients, fever is a critical warning sign, particularly in the presence of neutropenia (febrile neutropenia). Since wearables only measure skin temperature, an approximate threshold must be established.

1. Core Temperature: If the measurement persistently exceeds ∼37.8 °C, it is classified as fever according to [25].
2. Skin Temperature: Variations in skin temperature contribute to predicting fever. The selected smartwatch records nocturnal dermal temperature to detect future elevations. Thus, a sustained increase in skin temperature can anticipate imminent fever.

The determination of pyrexia is based on a combination of both sources. If the temperature exceeds the defined threshold (37.8 °C) or if the increase is greater than 0.4 °C for more than two hours, a fever alert is triggered.

### 3.4. Synthetic Data Generation

#### 3.4.1. Background

As stated in the introduction section, collecting a large amount of data from cancer patients is a major challenge. Therefore, although the final system will be evaluated with the data recorded from the 26 participants, the generation of synthetic data has been considered to create a robust model that has been trained and evaluated with diverse symptomatology.

To create a synthetic dataset, we considered the common adverse effects experienced by cancer patients. We generated a tabular database containing various patient profiles, which were then labeled by specialized clinical staff. The duration of the simulated measurements spanned 6 weeks.

To create a realistic longitudinal dataset simulating adverse effects in patients undergoing chemotherapy, we developed a rule-based parametric simulation model. This model generates data for a cohort of N=200 patients over a period of W=6 weeks, capturing not only the prevalence of individual symptoms but also their temporal evolution, inter-patient variability, and clinical correlations. As the participants in this study were treated with the inmunotherapy drug Pembrolizumab combined with chemotherapy, the adverse effects of this drug combination will be taken into account [36].

Regarding inter-patient heterogeneity, the model incorporates an individual susceptibility factor. Upon initialization, each simulated patient is assigned a unique factor drawn from a Gaussian distribution (μ=1.0, σ=0.20), bounded between 0.5 and 1.8. This factor scales all baseline symptom probabilities ($P_{base}$) for that patient throughout the simulation, representing their inherent propensity to develop adverse effects.

Clinical dependencies between symptoms were encoded using a set of correlation rules. These rules define “trigger–target” relationships. If a trigger symptom (e.g., Nausea) is present in a given week, the probability of its associated target symptom (e.g., Vomiting) is increased by a predefined additive value for that same week.

Table 2 collects the considered adverse effects, their prevalence, their correlation with other effects, and their simulation baseline probabilities $P_{base}$.

#### 3.4.2. Synthetic Data Generation Algorithm Formulation

Let $I=\{i_{1},i_{2},\ldots ,i_{N}\}$ be a set of *N* patients, $T=\{t_{1},t_{2},\ldots ,t_{W}\}$ a set of *W* weeks, and $S=\{s_{1},s_{2},\ldots ,s_{M}\}$ a set of *M* symptoms. The generation process for the synthetic dataset can be expressed as follows for each patient $i_{n}\in I$:

1. Assign the patient’s susceptibility factor $F_{sus}\left(i_{n}\right)$.
2. For each week $t_{k}\in T$:
  (a) For each symptom $s_{j}\in S$, compute the initial dynamic probability $P_{n,j,k}^{\prime }$:
    (2) $$P_{n,j,k}^{\prime }=P_{base}\left(s_{j}\right)\times F_{sus}\left(i_{n}\right)\times M_{temp}\left(s_{j},t_{k}\right)$$
    where $M_{temp}\left(s_{j},t_{k}\right)$ is the temporal modifier for symptom $s_{j}$ at week $t_{k}$.
  (b) Clamp the initial probabilities to the valid range [0, 1] to create a set of current probabilities for the week, $P_{current}$:
    (3) $$P_{current}\left(s_{j}\right)=\max\left(0,\min\left(1,P_{n,j,k}^{\prime }\right)\right)\;\forall s_{j}\in S$$
  (c) Iterate through the symptoms according to the predefined order $O=(s_{\left(1\right)},s_{\left(2\right)},\ldots ,s_{\left(M\right)})$. For each ordered symptom $s_{\left(j\right)}$ from j=1 to *M*:
    i. Draw a random number *u* from a uniform distribution u∼U(0,1). The state of the symptom is determined by:
      (4) $$X_{n,(j),k}=\left\{\begin{array}{cc} 1 & \mathrm{if}\;u<P_{current}\left(s_{\left(j\right)}\right)\\ 0 & \mathrm{otherwise} \end{array}\right.$$
    ii. If the symptom occurs ($X_{n,(j),k}=1$), immediately update the current probabilities for all its target symptoms. Let *C* be a set of correlation rules, where each rule is a tuple $\left(s_{trigger},s_{target},b\right)$ that adds a boost *b* to the probability of $s_{target}$ if $s_{trigger}$ occurs. For every rule $(s_{\left(j\right)},s_{target},b)\in C$:
      (5) $$P_{current}\left(s_{target}\right)\leftarrow \min\left(1,P_{current}\left(s_{target}\right)+b\right)$$

After iterating through all symptoms for the week, the vector $\left(X_{n,1,k},\ldots ,X_{n,M,k}\right)$ containing the final binary outcomes for patient $i_{n}$ at week $t_{k}$ is logged. This process is repeated for all patients and all weeks to generate the complete dataset.

### 3.5. Machine Learning Pipeline

**Figure 2:**
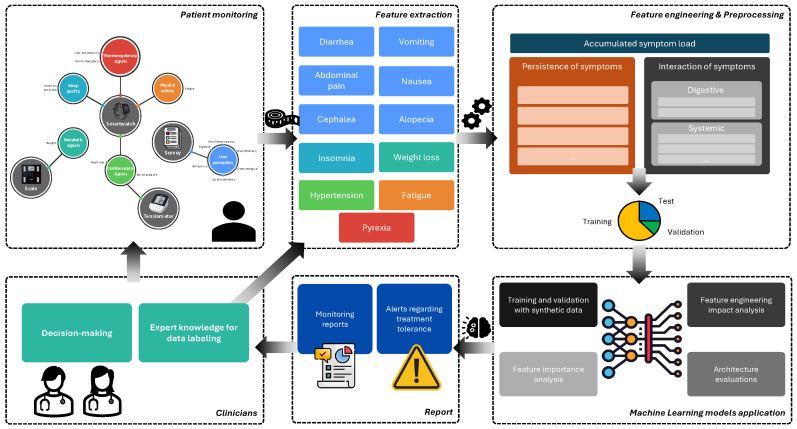
The proposed system for monitoring oncology patients consists of several stages. The first stage involves collecting physiological and self-reported data from the patients. Various features are then extracted from this data to train an ML model. The purpose of this model is to alert specialized clinical staff to significant changes that may require reassessment of the patient’s cancer treatment.

As introduced, the proposed system includes a tolerance assessment tool that leverages physiological and self-reported data using IoT devices. The ML pipeline consists of different stages, which are illustrated in Figure 2 and described in the following subsections.

#### 3.5.1. Data Labeling

To ensure clinical validity, the dataset was annotated by a Bienzobas medical specialist with expertise in oncology and supportive care. This expert-driven annotation process ensures that the binary features extracted from physiological signals and survey responses represent clinically meaningful outcomes. The expert in charge is a nurse specialized in the management of oncology patient cases and involved in patient-centered quality improvement initiatives and research.

The target label “low tolerance to current oncological treatment” was operationally defined by the clinical oncology team at Bienzobas based on explicit clinical criteria and validated against the judgment of the prescribing physician responsible for each patient. A patient was considered to present low treatment tolerance when at least one of the following conditions was met: the occurrence of adverse effects of grade ≥ 2 according to CTCAE criteria (Common Terminology Criteria for Adverse Events) requiring treatment modification; the need for dose reduction or delay in cycle administration; temporary or permanent treatment suspension due to toxicity; or unplanned hospitalization attributable to adverse effects of oncological treatment. Additionally, the clinical experience of the expert team allowed certain combinations of adverse effects to be considered indicative of low tolerance, even when individually they did not reach the aforementioned threshold, thus reflecting the complexity of real-world clinical assessment in oncology.

The labeling process was predominantly retrospective, based on the systematic review of patient medical records by the clinical oncology team at Bienzobas. In cases where access to updated medical records was available during the active period of the trial, labeling was carried out prospectively, allowing a more immediate assessment of the patient’s clinical status. In all cases, the assigned label was validated against the real clinical observation documented by the prescribing physician in charge of the treatment, ensuring that the binary label reflects a clinical decision grounded in real healthcare practice rather than an arbitrary categorization.

The validity of the labeling process is ensured by several complementary mechanisms that, taken together, provide methodological robustness equivalent to formal inter-rater agreement measurement. First, the operational definition of “low tolerance” is grounded in explicit and objective clinical criteria—adverse effects of grade ≥ 2 according to CTCAE, dose reductions, delays in treatment administration, suspensions and unplanned hospitalizations—which structurally delimits the annotator’s decision space and minimizes the subjective variability inherent in less systematized labeling processes. Second, the review was conducted by a clinical oncology team with proven expertise, which implicitly incorporates a process of expert deliberation and consensus acting as a quality control mechanism for the labeling. Finally, and most critically, each assigned label was validated against the clinical judgment of the prescribing physician responsible for the patient, who, as the professional with full access to the medical record and direct knowledge of the treatment course, constitutes the most clinically relevant reference standard in this context. This correspondence between the label and the actual therapeutic decisions documented in the medical record endows the labeling with an ecological validity that surpasses what a formal inter-rater agreement index between independent observers could guarantee on its own.

The binary label directly corresponds to a viable clinical decision: an observation labeled as “low tolerance” implies that the patient would have required, or effectively did require, a reassessment of the current therapeutic regimen. This correspondence between the label and the derived clinical action gives the predictive model direct practical utility, as its activation in a real clinical setting would signal the need for treatment review by the medical team.

Regarding the synthetic data, the labeling of these data was carried out retrospectively by the same clinical oncology team at Bienzobas that annotated the real patient data, applying the same operational criteria described above, thus ensuring methodological consistency between both datasets.

#### 3.5.2. Feature Engineering

In the first stage of the ML pipeline, feature engineering was applied to the original dataset to create new features from the weekly-reported adverse effects to capture the dynamic behavior and correlations between symptoms. In total, 19 new features were generated, resulting in a set of 30 input features for the models. These new features include:

- **Symptom load:**
  – Current Weekly Symptom Count. A quantitative measure representing the total count of distinct symptoms present in the current week.
  – Cumulative Symptom Load. The cumulative count of all symptoms reported by the patient from the beginning of the observation period up to the current week.
- **Symptom duration.** Duration-based variables were created to quantify the number of consecutive weeks a specific symptom has been active, such as the duration of fatigue.
- **Clinically relevant interactions.** Binary interaction variables were implemented to signal the co-occurrence of clinically related symptom clusters. These include:
  – A digestive interaction feature, activated by the simultaneous presence of nausea, vomiting, and diarrhea.
  – A systemic interaction feature, activated by the co-occurrence of fatigue and weight loss.
  – A neurological interaction feature, activated by the co-occurrence of headache and hypertension.

#### 3.5.3. ML Models

The clinical decision-making problem of whether to issue an alert for the reassessment of an oncological treatment can be formulated as a classic binary classification task $\hat{y}=f\left(x_{t}\right)$, such that $\hat{y}\approx y,\forall x\in X$, where *x* is the input vector from the input dataset *X*, *y* is the ground-truth label, and $\hat{y}$ is inference of the model. In this paradigm, a predictive model is trained to categorize each observation (e.g., a patient’s weekly status) into one of two discrete classes, thereby automating or assisting in the identification of patients who may require a treatment change.

To address this classification problem, several ML algorithms were considered:

- **Logistic Regression (LR)** [37]. This statistical model predicts a probabilistic value between 0 and 1, which is then mapped to one of the two classes based on a predetermined threshold. A key advantage of Logistic Regression is its high interpretability, as the model’s coefficients provide a clear indication of the influence of each input feature on the outcome.
- **Random Forest (RF)** [38]. An ensemble learning method that constructs a multitude of decision trees during training and outputs the class that is the mode of the classes of the individual trees. RFs generally exhibit high accuracy and are more robust against overfitting than single decision trees. Their main limitation is a reduction in direct interpretability, often requiring post hoc methods for model explanation.
- **Gradient Boosting Machines (GBMs)** [39]. This is a sequential ensemble technique that builds a robust predictive model by iteratively combining multiple weak learners, typically decision trees. Each successive model is trained to correct the residual errors of its predecessors, employing a gradient-based optimization approach to minimize the loss function. This boosting process enables GBMs to achieve state-of-the-art accuracy, making them a powerful tool for predictive analysis.
- **Support Vector Machines (SVMs)** [40]. This algorithm focuses on finding the optimal hyperplane that maximizes the margin of separation between the different data classes. This margin is defined by the data points closest to the hyperplane, known as support vectors. To handle non-linearly separable data, SVMs utilize a kernel to map data into a higher-dimensional space where linear separation is possible, rendering them a versatile and powerful classification tool.

The ML models were implemented using scikit-learn [41], and hyperparameters of the models were tuned to achieve the best performance.

#### 3.5.4. Data Preprocessing for Model Training

After feature engineering, both synthetic and real patient datasets were separated to evaluate the trained ML models. First, a patient-wise splitting procedure was performed: 30% of the users (real and synthetic) of each dataset were randomly separated to evaluate the models with users who were not involved in the learning process of the ML models (test users). Data from the other 70% of users were separated into two different subsets: 80% of the data were used for training the model (training dataset) and 20% were used as the validation dataset. After data splitting, the datasets are composed as follows:

- Training dataset: 672 observations from 140 synthetic patients and 121 observations from 18 real patients.
- Validation dataset: 168 observations from 140 synthetic patients and 78 observations from 18 real patients.
- Test dataset: 360 observations from 60 synthetic patients and 58 observations from 8 real patients.

In addition, a MinMax scaling was applied to all input features to normalize their values such that x∈[0,1]. This step is essential to ensure that all inputs are within the same range of values and that none dominate the learning process due to their scale [42].

### 3.6. Data Analysis

An Exploratory Data Analysis (EDA) was conducted regarding the data collected from the participant oncological patients. First, an analysis concerning reported adverse effects was carried out to evaluate the prevalence level compared to the prevalence rates reported in Table 2. In this context, the Phi correlation [43] was used to analyze the relationship between the reported effects. Additionally, the Chi-squared test was conducted to assess whether the combined occurrence of two adverse effects was statistically significant (*p*-value < 0.05).

Concerning the output of the LR, RF, and GBM $\hat{y}\in \left[0,1\right]$, a true classification value is considered when $\hat{y}>t_{C},\;t_{C}=0.5$. The performance of the ML models was evaluated using diverse metrics, including accuracy, precision, recall, specificity, F1-Score, and AUC-ROC.

Furthermore, due to the small sample size of participants recruited for this feasibility study, the practical use of the synthetic dataset was evaluated. Data from 18 participants (real users from the training dataset) were used to train an ML model that was evaluated with the synthetic dataset. This evaluation served as a cross-validation step to verify whether the patterns learned from the real-world cohort would apply to users who present a prevalence of adverse effects similar to those reported (see Table 2).

## 4. Results

### 4.1. Exploratory Data Analysis

**Figure 3:**
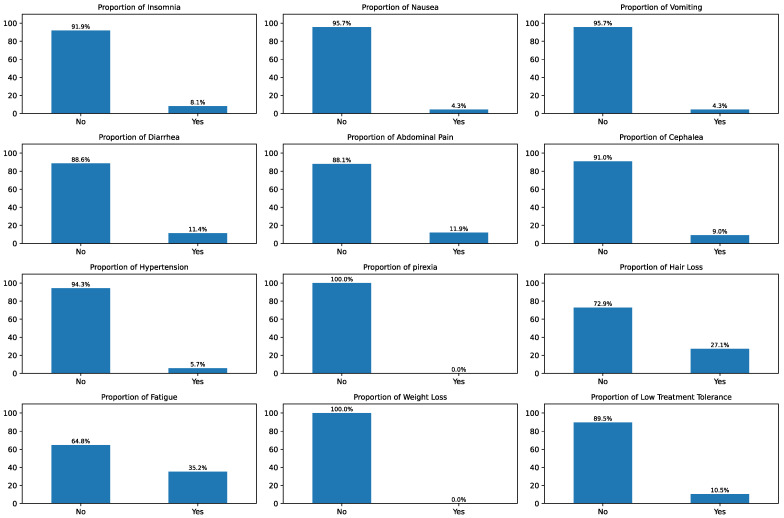
Univariate analysis of the adverse effects reported by the participating oncological patients.

An analysis of the adverse effects reported by oncological patients was performed. These results are summarized in Figure 3. This analysis identified several levels of prevalence among the symptoms observed. The most common symptoms were fatigue, reported in 35.2% of the cases, and hair loss, which occurred in 27.1% of the cases. A secondary group of symptoms exhibited a moderate incidence, affecting approximately one in ten patients. These included abdominal pain (11.9%), diarrhea (11.4%), headache (9.0%), and insomnia (8.1%). Other adverse effects were less common, with hypertension recorded in 5.7% of cases. Nausea and vomiting were the least frequently reported symptoms, each occurring in 4.3% of the records. It is important to note that neither pyrexia nor weight loss was reported, leading to a prevalence of 0.0% for these symptoms.

**Figure 4:**
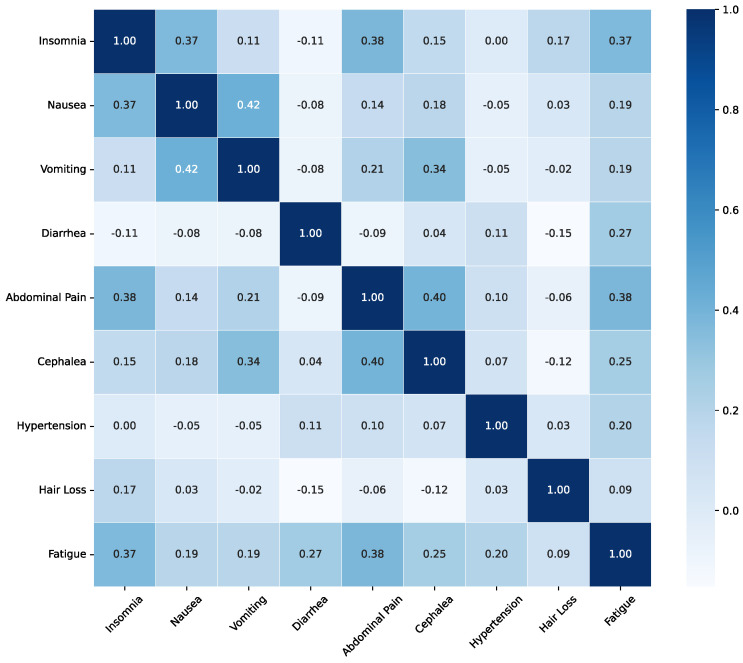
Phi correlation matrix between reported adverse effects in the participating oncological patients.

Figure 4 represents the Phi (ϕ) correlation results between adverse effects reported by the participants as a correlation matrix. Several clusters of co-occurring symptoms are apparent. Notably, a strong positive correlation is observed between Nausea and Vomiting. Similarly, Insomnia shows strong positive correlations with Fatigue and Abdominal Pain. Other notable positive correlations include Vomiting with Cephalea and Abdominal Pain, as well as Nausea with Fatigue.

To validate the statistical significance of the associations between these categorical variables, a Chi-squared test of independence was performed. The results for statistically significant pairs are presented in Table 3. The analysis confirms several statistically significant relationships. A highly significant association (p<0.0001) was found between Insomnia and Nausea, Insomnia and Abdominal Pain, Insomnia and Fatigue, Nausea and Vomiting, and Vomiting and Cephalea. Furthermore, other pairs also demonstrated a statistically significant association, including Vomiting and Abdominal Pain (p=0.0106), Nausea and Fatigue (p=0.0176), Vomiting and Fatigue (p=0.0176), Insomnia and Hair Loss (p=0.0271), and Nausea and Cephalea (p=0.0453).

As previously mentioned, the real-patient cohort dataset was completed with a rule-based synthetic dataset (see Section 3.4). The proportions of the adverse effects and the ground truth values are presented in Table 4.

### 4.2. ML Models’ Evaluation

**Figure 5:**
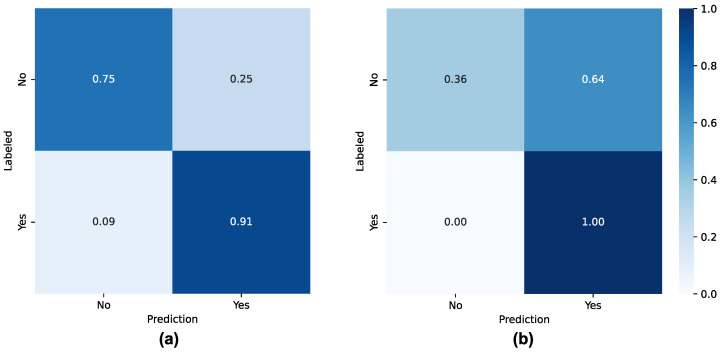
Confusion matrices obtained with the GBM model for (**a**) complete test dataset and (**b**) real-patient data from the test dataset.

The four trained ML models’ evaluation results on the test dataset are summarized in Table 5. The GBM model achieves the highest performance regarding accuracy (85%), precision (87%), recall (91%), specificity (75%), F1-Score (89%), and AUC-ROC (83%). Further analysis was performed with real-patient data using the GBM model. Table 6 details the GB evaluation using real-patient data from the test dataset. In addition, Figure 5 presents the confusion matrices obtained when using the GB model with the test dataset and only real-patient data. A decrease in GBM performance can be observed in accuracy (48%), precision (27%), specificity (36%), F1-Score (42%), and AUC-ROC (68%). However, the GBM detects all cases where an alert needs to be issued (recall: 100%).

**Figure 6:**
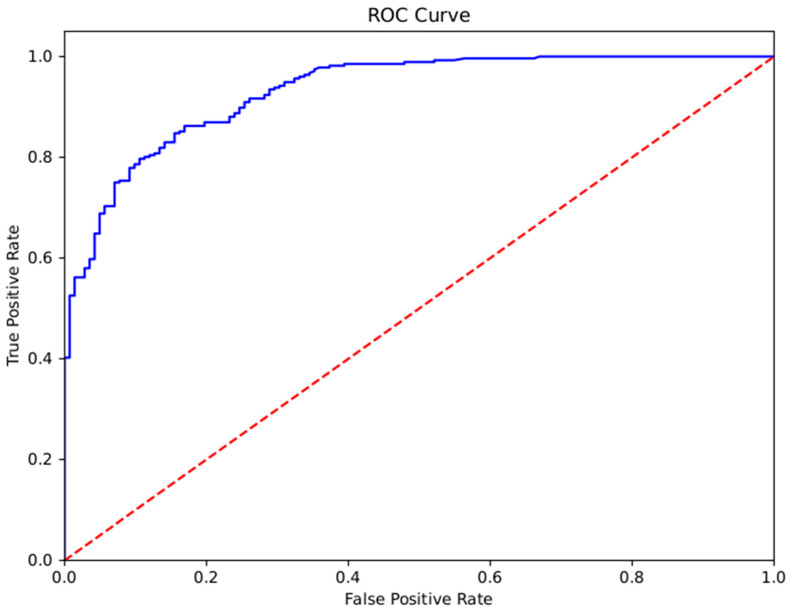
Representation of the ROC curve obtained using the GBM model with the test dataset.

**Figure 7:**
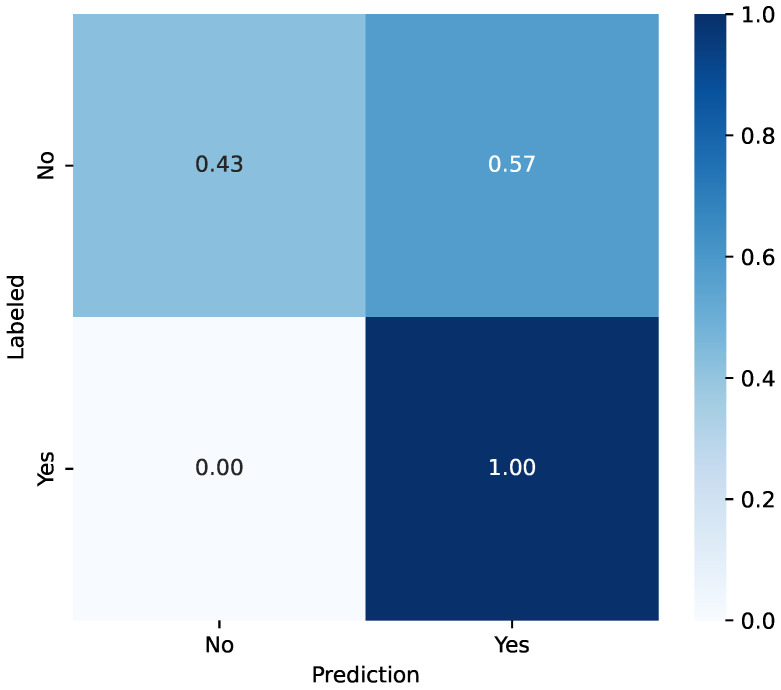
Confusion matrix obtained with the GB model ($t_{C}=0.6$) using real-patient data from the test dataset.

Concerning the high false-positive rate and the ROC curve (Figure 6), the model performance according to the value of $t_{C}$ was evaluated. Table 7 summarizes the GBM classification performance for diverse values of $t_{C}$. At lower thresholds (0.1–0.6), the model achieved perfect recall (1.00) at the cost of lower precision and specificity. As the threshold increased, accuracy and specificity improved while recall decreased. A $t_{C}=0.6$ was identified as the optimal choice, as it maximized both the F1-Score (0.45) and the AUC-ROC (0.71), providing a better balance between identifying positive cases and overall model performance. With this set $t_{C}$, the confusion matrix with the real-patient test dataset is presented in Figure 7.

### 4.3. Impact of Synthetic Dataset

The GBM model was trained with the real users from the training dataset and tested against the synthetic dataset to evaluate whether its use has practical benefits. As shown in Table 8, this evaluation yielded an accuracy of 0.56 and an AUC-ROC of 0.63. Notably, the model achieved a high precision of 0.85, indicating that when the model identified a tolerance issue based on real-world patterns, it was highly likely to be a true positive within the synthetic distribution. However, the recall was more moderate (0.49), suggesting that the specific manifestations of adverse effects in the pilot cohort only captured a subset of the broader range of toxicities represented in the synthetic population.

## 5. Discussion

This work presents an intelligent RPM system that attempts to improve the QoL of oncological patients. Specifically, this system aims to assess treatment tolerance by recording diverse physiological signals during daily-life scenarios together with self-reported adverse effects of the oncological patient. Previous studies have shown the effectiveness of these systems for RPM in oncology (see Section 2). However, our system’s main innovation is its unique integration of continuous, multi-source physiological data from a user-friendly IoT kit, combined with structured, patient-reported outcomes related to treatment side effects. By merging these objective and subjective data streams, our platform moves beyond analyzing individual metrics, allowing for a comprehensive, data-driven assessment of treatment tolerance. This is a critical aspect of oncological care that has been overlooked in earlier RPM implementations.

With this purpose, patients are provided with diverse IoT devices, including a BP monitor, a weighing scale, a smartwatch, and a smartphone. As presented in Figure 1, an easy-to-use Android app has been developed in the context of the project. This app issues a daily notification to remind patients when to perform the physiological measurements (e.g., weight, BP), guiding them through the process with diverse videos. In addition, the daily surveys allow self-reported health information to be captured from the patients. With the information collected from the physiological signals and surveys, diverse metrics are applied to detect 11 common oncological adverse effects.

An EDA was initially performed with data collected from 26 participants under oncological treatment who used the presented RPM system. A univariate analysis was initially performed to evaluate the prevalence of the adverse effects reported by patients (Figure 3). Fatigue (35.2%) and hair loss (27.1%) were the most prevalent symptoms. A secondary group of moderately frequent effects—including abdominal pain, diarrhea, headache, and insomnia—affected roughly one in ten patients. Less common symptoms included hypertension (5.7%), while nausea and vomiting were the least frequent at 4.3% each. No cases of pyrexia or weight loss were reported. This analysis revealed a significantly lower prevalence of adverse effects compared to clinical studies [36], which could be due to the reduced study sample size.

The correlation between adverse effects was thoroughly evaluated in this study. The Chi-squared analysis indicated that adverse effects experienced by oncological participants were consistent with the commonly reported treatment-related side effects (see Table 2). The Chi-squared analysis further supports the diversity of adverse effect pairings documented in the study, which enhances the credibility of the findings, despite the small sample size. For example, a very strong correlation was identified between nausea and vomiting (ϕ = 0.42, $\chi^{2}$ = *p* < 0.0001), reflecting a well-established clinical relationship. Additionally, a strong correlation was noted between insomnia and fatigue (0.37, *p* < 0.0001), aligning with information presented in the prevalence table. Furthermore, significant correlations were found within the headache cluster, linking headache to nausea, vomiting, and fatigue, all of which are documented associations. It is important to highlight that additional correlations not reported in Table 2 were also observed. For instance, a very strong correlation between abdominal pain and headache was found (0.40, *p* < 0.0001), suggesting a potential systemic response where inflammation or patient distress may manifest as pain in multiple areas. Moreover, a strong correlation between insomnia and hair loss was discovered (0.17, *p* = 0.0271), which may indicate shared underlying stressors or physiological pathways. Finally, the analysis demonstrated connections between insomnia and gastrointestinal distress, showing very strong correlations with nausea (0.37, *p* < 0.0001) and abdominal pain (0.38, *p* < 0.0001). This suggests that sleep disruptions could be a key indicator or contributing factor to gastrointestinal issues in patients.

Given that a higher prevalence of the adverse effects was expected with the correlations found in the previous analysis, we considered that the collected data were not sufficient to develop a robust ML that can issue an alert regarding low tolerance to current treatment. In order to solve this, a rule-based parametric model was proposed (Section 3.4). This model takes into account the demonstrated prevalence of adverse effects in patients treated with the immunotherapy drug Pembrolizumab and chemotherapy (Table 2). A synthetic dataset of 200 patients was generated with observations made over 6 weeks. The resulting dataset with 1200 observations was manually labeled by an experienced clinician, indicating whether the patient has low tolerance to the oncological treatment. In the same way, the data collected from the 26 participants was also labeled.

These labeled datasets were then used to train diverse ML models to issue an alert to clinicians regarding inferred low tolerance to the oncological treatment. Diverse ML models were trained and compared according to different binary classification evaluation metrics. Concerning the results, the trained GBM model achieves the highest performance with the test dataset, with an accuracy of 85%, a precision of 87%, a recall of 91%, a specificity of 75%, an F1-Score of 89%, and AUC-ROC of 83%. However, when evaluating the performance of the GBM with data collected from the participants, the performance decays for all evaluation metrics except for recall (100%). Although all cases that needed an alert to be issued were identified, this high recall had a high cost in terms of false-positive rates (64%, Figure 5b).

As a solution, regarding the representation of the ROC curve (Figure 6), the GBM model performance was evaluated according to the classification threshold $t_{C}$. The presented results detail the performance of the classification model across a range of discrimination thresholds from 0.1 to 0.9. A clear trade-off is evident between sensitivity and other performance metrics. At lower thresholds (0.1 to 0.6), the model achieves perfect recall (1.00), correctly identifying all positive instances. However, this is accompanied by lower precision (0.26–0.29) and specificity (0.32–0.43). As the threshold is increased, there is a corresponding decrease in recall but a general improvement in accuracy and specificity, with both reaching their peaks at a threshold of 0.9 (0.60 and 0.57). The F1-Score reaches its maximum value of 0.45 at a threshold of 0.6. Notably, the AUC-ROC also peaks at this threshold with a value of 0.71. Therefore, a threshold of 0.6 appears to offer the most balanced and optimal performance for this model, as it provides an effective trade-off between the need to identify positive cases and the overall predictive power. From a clinical perspective, prioritizing a 100% recall ensures that no high-risk patient is overlooked by the system, which is paramount for patient safety during oncological treatment. However, the current false-positive rate (57%) at this threshold imposes a substantial alert burden on healthcare professionals. While the current system successfully serves as a sensitive screening layer, the next phase of this project must focus on refining model specificity. Reducing unnecessary interventions through more granular data processing will be essential to making the system sustainable for long-term clinical workflows.

Furthermore, the benefits of incorporating the synthetic data into the ML pipeline have been analyzed. With this purpose, the GBM model was trained using only the real-patient data from the training dataset, and evaluated with the synthetic dataset to demonstrate how a model trained on a limited, low-prevalence sample struggles to generalize to the broader clinical reality. As shown in Table 8, when the model is trained on real-world cases, it achieves an accuracy of 0.56, a precision of 0.85, and a recall of 0.49. The low recall suggests that the real-world participants exhibited a narrower spectrum of symptoms than what is reported in larger oncological studies. By using the synthetic data to fill these gaps in event prevalence, we enable the model to recognize high-risk patterns that were not present in our initial small sample size, thereby moving toward a more robust and safety-oriented screening tool that prioritizes high recall.

Concerning the results, we consider the proposed system a clinically viable tool that can effectively flag high-risk patients. However, several limitations of this study should be acknowledged. The primary limitation of this work is the reduced sample size of participants undergoing oncological treatment (26 patients). Specifically, the frequency of adverse effects reported by the recruited participants was lower than the reported clinical prevalence. This discrepancy in data distribution necessitated the generation of a synthetic dataset to ensure the ML models were exposed to a sufficient variety of clinical scenarios. While the use of synthetic data allowed for the training of a robust model capable of achieving high recall, we acknowledge that this approach introduces specific challenges regarding the reliability and interpretability of the findings since it may not fully capture the complex, non-linear biological interactions inherent in real patient physiology. Furthermore, the study’s duration was limited, preventing the analysis of long-term trends in treatment tolerance. The patient-reported outcomes, while valuable, are also subject to recall bias and subjective interpretation. Despite these constraints, this work serves as a vital proof-of-concept. It demonstrates the potential of an integrated RPM system in oncology while highlighting that future iterations must prioritize the collection of larger, independent clinical datasets to reduce reliance on synthetic augmentation and further refine the model’s clinical precision.

## 6. Conclusions

In this work, we successfully designed and implemented an intelligent RPM system aimed at improving the quality of life of oncological patients by assessing their tolerance to treatment. The system integrates objective physiological data from a user-friendly IoT kit with subjective, patient-reported outcomes. While the initial real-world data from 26 participants was insufficient for robust model training, it revealed significant symptom correlations that informed the development of a larger, clinically grounded synthetic dataset.

The core achievement of this study was the development of a GBM model capable of identifying both synthetic and real patients with low treatment tolerance with 85% accuracy, 91% recall, and 89% F1-Score. Although the model’s performance was negatively impacted by the lower prevalence of adverse effects reported by patients, increasing the sample size is expected to better align the distributions of these adverse effects with those observed in clinical studies. This enhancement in sample size should improve the model’s performance, making it comparable to that observed using the synthetic dataset.

## Figures and Tables

**Table 1:** Relation between biological variables, health domain and extracting device.

Variable	Health Domain	Source
Sleep-Log	Sleep quality	Smartwatch
Daily Activity	Physical activity	Smartwatch
Skin temperature	Thermoregulatory signals	Smartwatch
Heart Rate	Cardiovascular signals	Smartwatch, Tensiometer
Blood Pressure	Cardiovascular signals	Tensiometer
Weight	Metabolic signals	Scale
Diarrhea, Vomiting, Abdominal pain, Nausea	Digestive	Survey
Alopecia	Dermatological	Survey

**Table 2:** Prevalence of adverse effects in patients treated with Pembrolizumab and chemotherapy [36].

Adverse Effect	Prevalence (%)	Correlated with	P_base_ (%)
Insomnia	21	Fatigue	21
Nausea	40–67	-	53.5
Vomiting	24–34	Nausea	29
Diarrhea	31–55	-	43
Abdominal pain	24–34	Diarrhea	29
Cephalea	20–30	Fatigue, Nausea, Vomiting	25
Hypertension	33–68	-	45.4
Pyrexia	20–28	Fatigue	24
Alopecia	47–61	-	54
Fatigue	47–70	-	58.5
Weight loss	20–34	Nausea, Vomiting, Diarrhea	27

**Table 3:** Chi-squared test significant results for the appearance of adverse effects in participating oncological patients.

Adverse Effect 1	Adverse Effect 2	*p*-Value
Insomnia	Nausea	<0.0001
Insomnia	Abdominal Pain	<0.0001
Insomnia	Hair Loss	0.0271
Insomnia	Fatigue	<0.0001
Nausea	Vomiting	<0.0001
Nausea	Cephalea	0.0453
Nausea	Fatigue	0.0176
Vomiting	Abdominal Pain	0.0106
Vomiting	Cephalea	<0.0001
Vomiting	Fatigue	0.0176
Diarrhea	Fatigue	0.0003
Abdominal Pain	Cephalea	<0.0001
Abdominal Pain	Fatigue	<0.0001
Cephalea	Fatigue	0.0006
Hypertension	Fatigue	0.0079

**Table 4:** Data distribution of the joint dataset (real-patient and synthetic data).

Adverse Effect	No (%)	Yes (%)
Insomnia	58.7	41.3
Nausea	40.5	59.5
Vomiting	42.6	57.4
Diarrhea	48.9	51.1
Abdominal Pain	51.4	48.6
Cephalea	62.9	37.1
Hypertension	44.4	55.6
Pyrexia	74.8	25.2
Hair Loss	42.8	57.2
Fatigue	24.7	75.3
Weight Loss	52.6	47.4
Low Treatment Tolerance	36.6	63.4

**Table 5:** Performance comparison of the trained ML models using the test dataset.

Model	Accuracy	Precision	Recall	Specificity	F1-Score	AUC-ROC
LR	0.84	0.86	0.91	0.70	0.88	0.81
SVM	0.81	0.84	0.89	0.66	0.86	0.77
RF	0.83	0.85	0.90	0.69	0.87	0.79
GBM	0.85	0.87	0.91	0.75	0.89	0.83

**Table 6:** Performance of the GBM model evaluated with real-patient and synthetic data (test dataset).

Data	Accuracy	Precision	Recall	Specificity	F1-Score	AUC-ROC
Real	0.48	0.27	1.0	0.36	0.42	0.68
Synthetic	0.84	0.87	0.92	0.62	0.89	0.77

**Table 7:** GBM model evaluation results with the real patient test dataset according to the classification threshold.

Threshold	Accuracy	Precision	Recall	Specificity	F1-Score	AUC-ROC
0.1	0.45	0.26	1.00	0.32	0.41	0.66
0.2	0.45	0.26	1.00	0.32	0.41	0.66
0.3	0.47	0.26	1.00	0.34	0.42	0.67
0.4	0.47	0.26	1.00	0.34	0.42	0.67
0.5	0.48	0.27	1.00	0.36	0.42	0.68
0.6	0.53	0.29	1.00	0.43	0.45	0.71
0.7	0.55	0.29	0.91	0.47	0.43	0.69
0.8	0.53	0.26	0.82	0.47	0.40	0.64
0.9	0.60	0.29	0.73	0.57	0.41	0.65

**Table 8:** Performance of the GBM model evaluated with the synthetic dataset when it is trained using real-patient data.

Model	Accuracy	Precision	Recall	Specificity	F1-Score	AUC-ROC
GBM	0.56	0.85	0.49	0.76	0.63	0.63

## Data Availability

The datasets of the current study are available from the corresponding authors upon reasonable request.

## Abbreviations

The following abbreviations are used in this manuscript:

IoT	Internet of Things
RPM	Remote Patient Monitoring
Machine Learning	ML
ECG	Electrocardiogram
BP	Blood Pressure
HR	Heart Rate
LR	Logistic Regression
RF	Random Forest
GBM	Gradient Boosting Machine
SVM	Support Vector Machine
EDA	Exploratory Data Analysis
AUC	Area Under the Curve
PRO	Patient-Reported symptom monitoring
